# The Feeding of School Children

**Published:** 1911-08-05

**Authors:** 


### The Feeding of School Children.

The work of provision of meals for school children is intimately connected with the work of medical inspection; and unless it is regarded as a branch of the school medical service, it may degenerate into a system of doles and be administered in a haphazard fashion on merely sentimental lines. It is pointed out in the report on the working of the Education (Provision of Meals) Act, 1906, for the year ending March 31, 1910, that it is only by the administration of the Act as part of the school medical service, and by the scientific organisation of the work with due regard to medical and educational considerations, that wasteful and reckless expenditure can be prevented and full value, in the form of increased vigour and more effective education, be obtained for the expenditure of the authority. Even if it is impracticable, when the operations under the Act are on a large scale, for the school medical officer to examine personally all children recommended for meals, he can, in the opinion of the Board of Education, take a large part in the selection, and by concentrating on those cases which are doubtful and those in which actual neglect is suspected, he can do a great deal towards the establishment of a proper standard. He can give valuable advice in the choice of food, and test the results of different dietaries. If he visits the kitchens and dining centres from time to time he can see that satisfactory methods are adopted, and ensure, for instance, that especial attention is given to the more delicate children, and to those whom it is difficult to induce to eat the food provided. The Board has not yet received a sufficient number of the reports of school medical officers for the year under review to enable them to form an accurate opinion of the extent to which these officers have taken an active part in the organisation of the arrangements for the meals. It is, however, gathered from the returns that there were seventy cases in which the school medical officer had some share in the recommendation of the children, and thirty-nine cases in which recommendation was made by school nurses. In the previous year the numbers were fifty-five and thirty respectively; and it appears that there is a growing tendency to correlate the two functions of the local education authority and to take advantage of the expert opinion of the school medical officer in the selection of children for meals.

